# Advances in PBT Binder and Its Application in Propellants

**DOI:** 10.3390/polym17091151

**Published:** 2025-04-23

**Authors:** Ning Zhang, Xifei Gao, Yunjun Luo, Weihai Zhang, Yanping Xin, Kai Zhang, Chen Xue, Han Zhang, Jiao Wei, Hui Wang

**Affiliations:** 1 School of Materials, Beijing Institute of Technology, Beijing 100081, China; zhangning09106@163.com; 2 Xi’an North Huian Chemical Industry Co., Ltd., Xi’an 710302, China; gxfei9226@163.com (X.G.); xinyanpingxyp@163.com (Y.X.); xczhangkai2012@163.com (K.Z.); Xchen_vivian@163.com (C.X.); 16602925879@163.com (H.Z.); qqc5693@163.com (J.W.); wh1997071@163.com (H.W.); 3 Key Laboratory for Ministry of Education of High Energy Density Materials, Beijing 100081, China

**Keywords:** PBT binder, solid propellant, performance, application

## Abstract

3,3-Bis (azide methyl) oxy-butyl ring (BAMO)-tetrahydrofuran (THF) copolyethers (PBT) are some of the most promising energetic binders. In this paper, the methods of synthesis of PBT binders are reviewed, and the research progress in PBT binders and PBT-based solid propellants in terms of their thermal and combustion behavior, curing and rheology properties, energy and aging properties, and mechanical and safety performances are systematically summarized. The problems and shortcomings of PBT binders in the application of solid propellants and their thriving trends are pointed out, providing support for speeding up the practical application of PBT binders in high-energy solid propellants.

## 1. Introduction

With the improvement of solid propellant technology, high-energy, insensitive, low-characteristic signal and pollution-free propellants are becoming the development direction of solid propellants in the future. To improve the energy properties of solid rocket propellants and give consideration to their mechanical, technological, and combustion properties, there exists an imperative to develop advanced energetic binders as replacements for conventional inert binder systems such as polyurethane (PU) and hydroxyl-terminated polybutadiene (HTPB) [1,2,3,4,5,6,7]. This fundamental transition underscores the paramount significance of exploring innovative energetic binder architectures in modern propellant engineering [8,9].

The evolutionary trajectory of energetic binders constitutes a pivotal component in the advancement of solid rocket propulsion technology. These specialized binders not only function as binder matrices but also serve as intrinsic energy reservoirs through their chemical architecture, thereby enhancing the specific impulse and combustion efficiency in propellant formulations. Historical records indicate that systematic investigation into energetic binders commenced in the mid-1960s, when propellant chemists worldwide initiated the strategic incorporation of energetic functional groups into conventional polymer side chains. Key moieties under exploration included nitro (-NO_2_), nitrate ester (-ONO_2_), azido (-N_3_), difluoroamino (-NF_2_), and fluorodinitro (-CF(NO_2_)_2_) groups [10,11].

The earliest representative of typical energetic binders was nitrocellulose (NC) [12,13], which is mainly used for double-base propellants, but its disadvantage is that it is brittle. Subsequently, polyether polyurethane (PEPU) was studied [14,15], but due to the introduction of nitrate groups, although the energy was increased, the sensitivity was higher. Following extensive exploratory studies, research emphasis gradually converged on two principal categories: azido-functionalized polyether binders (including homopolymers and copolymers of GAP, BAMO, and AMMO) [16,17,18,19] and nitrate ester binder (NEPE) systems [20,21,22]. Notably, 3,3-bis(azidomethyl)oxetane (BAMO) emerged as the azido monomer with the highest recorded heat of formation. However, its practical application is constrained by inherent sensitivity issues, necessitating homopolymerization to produce poly-BAMO (PBAMO) with improved safety profiles. The structural symmetry of azidomethyl groups in PBAMO induces semicrystalline behavior at ambient temperatures, characterized by an elevated glass transition temperature (*T_g_*) and compromised flexibility, rendering it unsuitable for direct propellant applications [23]. Therefore, a flexible polytetrahydrofuran (THF) segment is introduced by random copolymerization to reduce or eliminate the crystallization of PBAMO, thus obtaining flexible BAMO/THF copolyether (PBT), which ensures that the prepared high-energy propellant has admirable low-temperature mechanical properties and combustion performance [24,25]. Moreover, PBT binder is a viscous fluid at room temperature that has good compatibility with other components of propellants and good comprehensive properties, giving PBT-based propellants the advantages of a high specific impulse, clean gas, and so on; thus, PBT binder is an ideal energetic binder for preparing high-energy solid propellants [26]. Meanwhile, it can meet the requirements of having a low sensitivity and a low characteristic signal of the binder for solid propellants, leading to it becoming one of the hot spots in the field of solid propellants.

**Figure 1:**
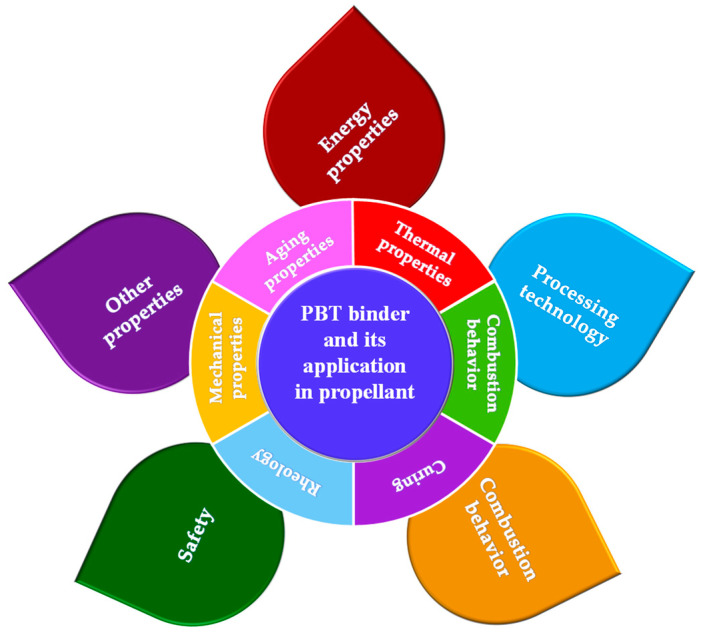
System architecture of PBT binder and propellant performance studies.

In this paper, the synthesis methods of PBT binders and their research status in terms of their thermal properties, combustion behavior, curing, rheology, mechanical properties, and aging properties are briefly described, and the research progress in PBT propellants in terms of their energy properties, processing technology, combustion behavior, safety, and other properties is summarized and analyzed (Figure 1), providing many new insights for accelerating the application of PBT binders in high-energy solid propellants.

## 2. Research on Synthesis and Properties of PBT Binders

### 2.1. Study on Synthesis of PBT Binder

**Figure 2:**
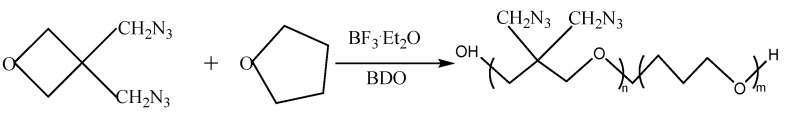
Synthesis diagram of PBT [27].

The synthesis of PBT, an azide-based copolyether binder, has been extensively investigated, with cationic ring-opening polymerization emerging as a typical representative. Manser [27] synthesized BAMO/THF copolyether (PBT) by cationic ring-opening polymerization with the ratio of 1:1. The specific chemical synthesis reaction is shown in Figure 2.

THF is employed as a co-binder to suppress the crystallinity of BAMO polymers. Concurrently, the researchers studied the monomer feeding strategies, polymerization method, different link ratios, molecular weight design and properties of BAMO and THF copolymerization. At present, the preparation of PBT is generally initiated with pentaerythritol, which forms a bis-substituted oxacyclobutane structure, and then it is substituted by azide SN_2_ to obtain a BAMO monomer [28,29]. Finally, PBT is obtained by cationic ring-opening polymerization with THF cation. Taking the copolymer with the molar ratio of BAMO/THF of 60:40 as an example, the physicochemical characteristics of PBT are shown in Table 1 [30].

**Figure 3:**
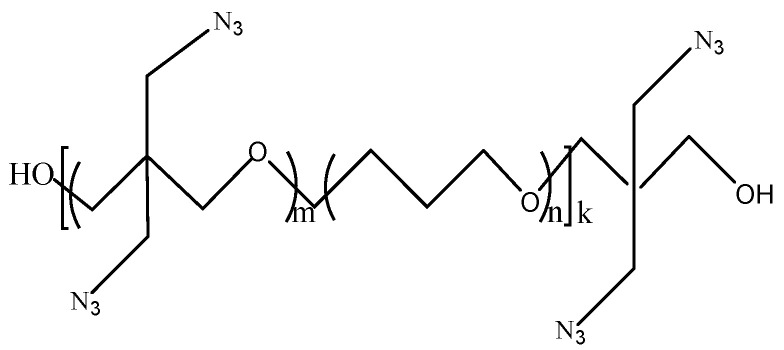
PBT of alternate multi-block structure [33].

The BAMO/THF azide copolymer (PBT) with a molar ratio of 50:50 was successfully synthesized using BF_3_·Et_2_O as the catalyst, achieving a reaction yield of 70%. The resulting polymer exhibited weight-average (Mw) and number-average (Mn) molecular weights of 4771 and 2900, respectively [31]. A scholar [32] used dichloromethane as a solvent to synthesize PBT by cationic ring-opening polymerization. The number-average relative molecular weight of PBT was about 1700~2800, and its viscosity basically met the regulatory requirements. With the increase in THF content, the melting point and glass transition temperature of PBT decreased rapidly, which significantly improved the low-temperature mechanical properties of PBT. This method lays a foundation for the subsequent research on the optimization of PBT binder preparation. Zheng et al. [32] demonstrated a strategic synthetic approach for poly (BAMO-co-THF) (PBT) binders through molecularly tailored Williamson etherification. Utilizing 3,3-bis(azidomethyl)oxetane homopolymer (PBAMO) and polytetrahydrofuran (PTHF) as precursors, the team engineered alternating multi-block architectures by precisely controlling stoichiometric ratios (BAMO: THF = 1:1–3:1). As shown in Figure 3, this methodology enabled microstructural regulation of the copolymer, producing distinct multi-segmented morphologies. The resulting azido-functionalized polyether binder exhibited exceptional structural tunability with its alternating blocks synergistically balancing energetic density (derived from PBAMO’s high azide content) and chain mobility (contributed by PTHF’s flexible oxybutylene units). Critically, this architecture endowed propellant formulations with enhanced mechanical resilience, addressing the inherent brittleness of conventional BAMO-based systems. The study underscores the efficacy of block copolymer design in reconciling energy-output requirements with processability constraints, establishing a foundational framework for advanced binder development in high-performance solid propellants.

The Liming Chemical Research Institute [34] developed a scalable synthesis methodology for azido-functionalized PBT polyethers via the controlled cationic ring-opening polymerization of BAMO and THF. By refining catalyst systems (BF_3_·Et_2_O coordination) and reaction kinetics, the team effectively mitigated critical safety challenges associated with BAMO scale-up synthesis, achieving precise molecular weight regulation through monomer feed ratio optimization and chain-transfer agent modulation. This methodology enabled the reproducible production of structurally uniform PBT binders with tailored chain architectures as validated by narrow polydispersity indices and quantitative azide group retention. The breakthrough establishes an industrially viable pathway for synthesizing high-purity energetic binders, addressing long-standing limitations in process safety and batch-to-batch consistency for propellant-grade PBT applications.

PBT binders generally possessed the disadvantages of high viscosity. Wang et al. [35] synthesized branched PBT (B-PBT) binders through the trifunctional initiator-mediated nucleophilic grafting of PBAMO/THF prepolymers. This architectural modification induced significant rheological improvements with dynamic viscosity reductions of 37% (15,000 vs. 23,828 mPa·s at 50 °C) and 34% (3428 vs. 5171 mPa·s at 80 °C) relative to linear PBT counterparts. The reduced chain entanglement from controlled branching density (1.5–2.3 branches/core) enhanced processability while maintaining azide functionality (>97% retention via FTIR). Thermal analysis revealed superior stability with dual decomposition stages: initial azide scission (Δ*H* = 1481 J·g^−1^) followed by backbone degradation above 300 °C. Mechanistically, the star-shaped topology balanced energy density (*Qv* = 5890 J/g) with mechanical compliance (*σ_m_* = 5.29 MPa, *ε_b_* = 516%), demonstrating how molecular architecture engineering can effectively decoupling the inherent energy-processability trade-off in energetic binder design.

### 2.2. Research on Properties of PBT Binder

Researchers have conducted in-depth investigations on the thermal properties, combustion performances, curing behavior, rheological properties, mechanical performances, and aging properties of PBT binders.

In the study of thermal and combustion properties of PBT binders, Toshio et al. [36] systematically elucidated the thermochemical decomposition pathways of PBAMO, BAMO/THF (60:40) copolyether, and crosslinked BAMO/THF systems through combined DTA-TG-IR analyses, and they proposed the thermal decomposition mechanism of BAMO/THF. Their work revealed a biphasic decomposition mechanism: (1) the rapid exothermic scission of azide (-N_3_) groups, releasing 35% mass loss via N_2_ evolution and forming imine (-CH=NH) intermediates, as confirmed by IR spectral shifts (2100→ 1650/3400 cm^−1^); (2) gradual carbon backbone degradation (>520 K) yielding residual char (65% mass loss). Crosslinked systems exhibited analogous N_3_ decomposition kinetics but with suppressed gas-phase reactivity due to network stabilization. Crucially, the primary exothermicity (Q = 1481–2460 kJ·kg^−1^ directly correlated with azide density, explaining the threefold burning rate enhancement observed when BAMO/THF molar ratios increased from 50:50 to 70:30. This mechanistic framework establishes azide scission as the rate-limiting step in combustion, providing critical insights for tailoring binder energy release profiles in solid propellants. Kubota et al. [37] studied the combustion performance of PBT and found that at a specific initial temperature, the burning rate increased linearly with the increase in pressure. At the same initial temperature and different BAMO/THF ratios (70:30, 60:40 and 50:50), the burning rate of PBT was c susceptible to the monomer ratio in the copolyethers. These results provide an important reference for applying PBT binders in propellants and emphasize the key effect of monomer ratio on their properties.

**Figure 4:**
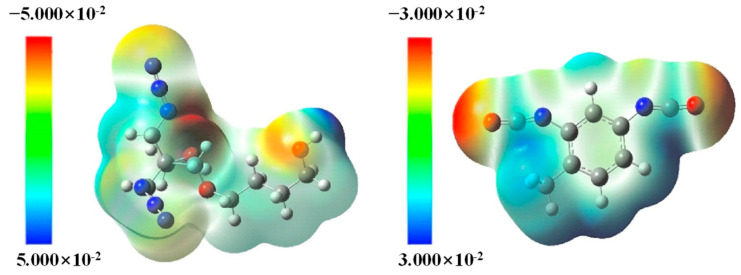
The molecular electrostatic potentials of PBT and 2,4-TDI [38].

**Figure 5:**
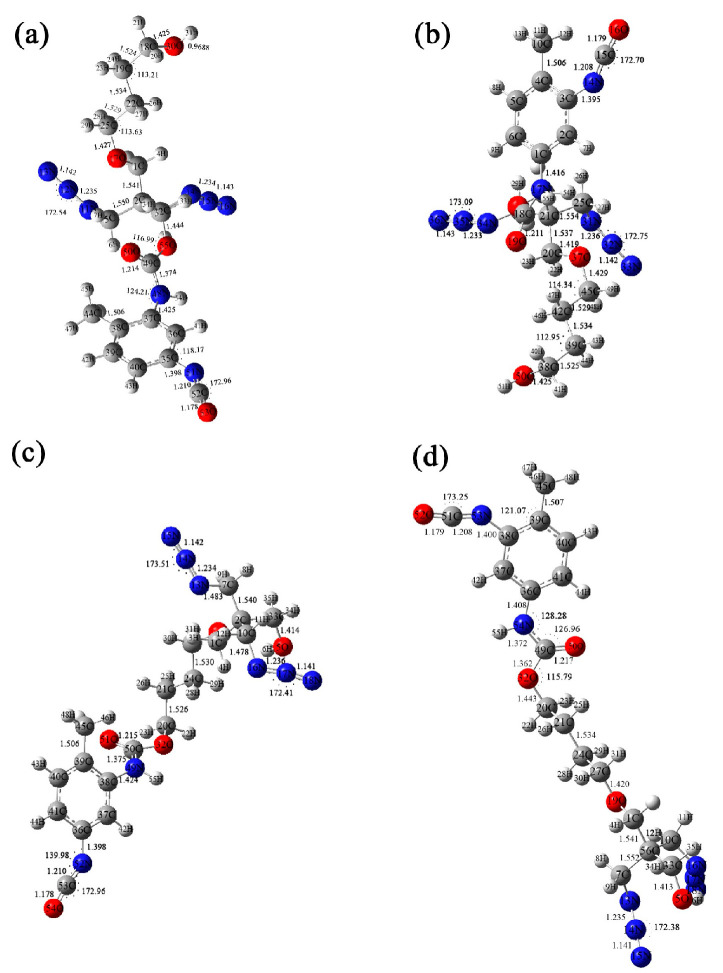
Configuration and parameters of four reaction products: PBT and (2,4) TDI-AC (**a**), PBT and (2,4) TDI-AD (**b**), PBT and (2,4) TDI-BC (**c**) and PBT and (2,4) TDI-BD (**d**) [38].

Regarding the curing characteristics of PBT binders, Chen et al. [38] studied the structure–property of model compounds of BAMO-THF copolyether (PBT) and curing agent (TDI) by using density functional theory (DFT), and they explored the possible reaction sites of curing reaction by calculating and analyzing the electrostatic potential maps (Figure 4). The results showed that there were many reaction sites between TDI and PBT. Based on the model compounds, when the ratio of PBT to 2,4-TDI was 1:1, the four thermodynamically feasible reaction products were AC, AD, BC and BD (Figure 5). Meanwhile, the thermodynamically stable structure of the curing reaction process was determined, and the transition state in the reaction path was calculated so as to determine the most likely curing reaction path and activation gibbs energy, which is of great significance for in-depth understanding of the interaction between the PBT binder and TDI curing agent and the curing mechanism.

**Figure 6:**
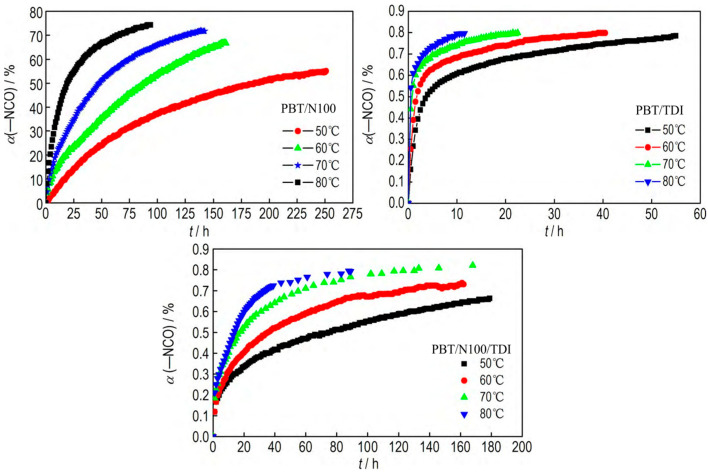
The α-t curves of -NCO in PBT/N-100, PBT/TDI, and PBT/N-100/TDI systems at different temperatures [39].

Li et al. [39] investigated the curing kinetics of three PBT-based binder systems (PBT/TDI, IPBT/N-100 and PBT/TDI/N-100) using Fourier transform infrared spectroscopy (Figure 6). The results demonstrated that all three systems followed second-order reaction kinetics throughout the curing process. In the PBT/TDI system, due to the difference of -NCO activity at different positions on TDI, the conversion rate of the PBT/TDI system was less than 60% in the first stage and more than 60% in the second stage. At identical temperatures, the first-stage reaction rate was significantly higher than that of the second stage with apparent activation energies of 54.71 kJ·mol^−1^ and 56.50 kJ·mol^−1^, respectively. The apparent activation energy of PBT/N-100 is 63.10 kJ·mol^−1^, which is higher than that of PBT/TDI, and the pre-exponential factor A is 1.63 × 107 h^−1^. It is worth noting that the PBT/TDI/N-100 combination system exhibits synergies: at the conversion stage < 65%, its apparent activation energy (71.17 kJ·mol^−1^) and pre-exponential factor (4.58 × 10 Guess h^−1^) are 28 to 37 times higher than those of single-component systems, respectively. This can be attributed to the synergistic interaction of the TDI chain extension reaction and N-100 crosslinking reaction forming multiple active sites. The kinetic analysis confirms that the curing dynamic behavior of PBT binders can be precisely controlled through curing agent selection, providing critical theoretical guidance for designing high-performance propellant binders.

In terms of the research on the curing rheological properties of PBT binders, Ren et al. [40] have carried out in-depth research on the PBT/N-100 bonding system. With the help of a rheometer, they comprehensively analyzed the change in modulus during curing and the change in viscosity before gelation. It is found that the isothermal curing process of the PBT/N-100 system shows the characteristics of a catalytic kinetic model. According to the dynamic change in modulus with time, the curing process can be clearly divided into three stages: reaction control, gelation and diffusion control. Further study shows that the viscous flow activation energy of the control stage is 36.271 kJ·mol^−1^, and the reaction activation energy is 54.882 kJ·mol^−1^. In addition, by introducing an Eyring model for calculation, the ideal curing conditions of the PBT/N-100 bonding system were determined: that is, the temperature was 60 °C and the time was about 160 h. This research result provides a key basis for in-depth understanding of the curing mechanism of PBT/N-100 bonding system. It also lays a solid theoretical foundation for optimizing the solidification process of propellants and improving the performance of propellants, and it effectively promotes the practical application and development of PBT binders in the field of propellants.

**Figure 7:**
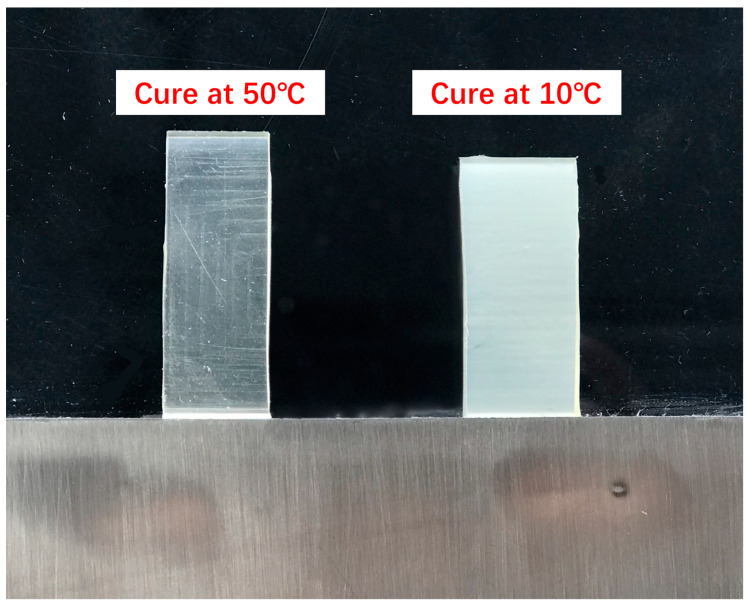
Appearance of PBT elastomer at different curing temperatures [41].

In recent studies on PBT binders, Zhao et al. [41] systematically investigated the effects of curing conditions and crosslinking parameters on material properties. By fixing the isocyanate-to-hydroxyl molar ratio (*R*-value) at 1.0, they observed that PBT elastomers cured at 10 °C exhibited significantly lower optical transparency than those cured at 50 °C (Figure 7), which was attributed to the retained microcrystalline domains formed under low-temperature curing. Furthermore, variations in the *R*-value (0.8–1.3) were found to critically influence network architecture and mechanical behavior. Increasing R-values led to enhanced crosslinking density, which improved tensile strength at both 20 °C and 60 °C but reduced elongation at break due to restricted chain mobility. Notably, the glass transition temperature (*T_g_*) of the elastomers decreased progressively with higher *R*-values, suggesting a trade-off between mechanical reinforcement and segmental flexibility. These findings highlight the tunability of PBT-based binders for propellant applications, where balanced strength and elasticity are essential to withstand extreme operational conditions while maintaining processability. This systematic investigation emphasizes the importance of optimizing curing parameters and stoichiometry to tailor PBT elastomers for advanced solid propulsion systems.

In the field of solid rocket propellants, PBT has been comprehensive investigated as a promising binder. To characterize its mechanical behavior, Li et al. [42] performed a systematic investigation of PBT/N-100 binder films, specifically examining the influence of the curing parameter *R*-value ([NCO]/[OH] molar ratio) on tensile properties. Under the same test temperature, when the *R*-value increases from 0.8 to 1.7, the fracture type of PBT/N-100 film remains unchanged, there is no yield point in the stress–strain curve, and high elastic deformation can occur under low stress, showing high elastic material properties. During this process, the mechanical properties of the film showed regular changes: the tensile strength *σ_m_* gradually increased from 0.902 MPa to 1.365 MPa, while the fracture elongation *ε_b_* decreased from 220% to 110% (Table 2). It shows that the change in *R* value has a significant effect on the mechanical properties of PBT/N-100 film, and this study has important guiding significance for the reasonable selection of curing parameters in the design of PBT propellant formulation.

**Figure 8:**
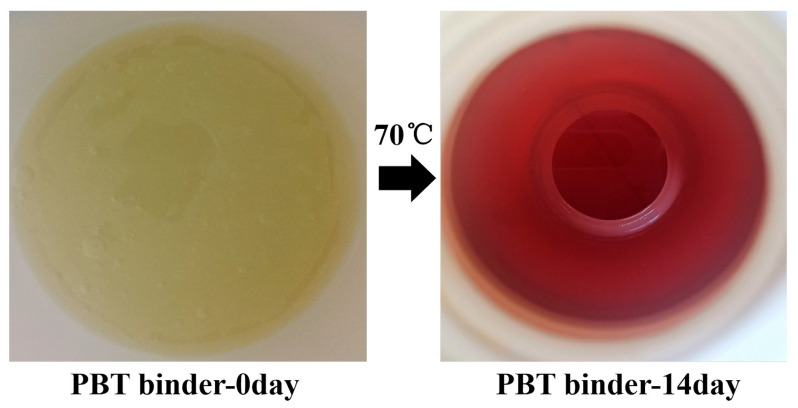
Appearance of PBT prepolymers at 70 °C for 14 days without aging on the (**left side**) and aging on the (**right side**) [43].

**Figure 9:**
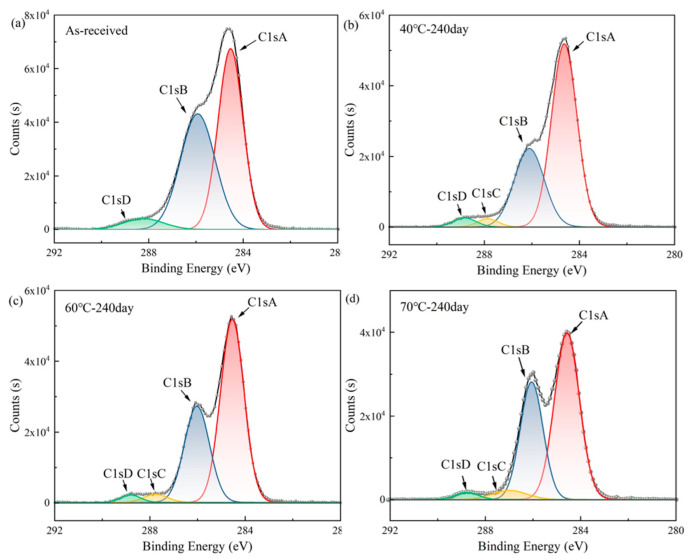
C1s spectra of PBT elastomer at different aging times [43]: (**a**) original sample; (**b**) age at 40 °C for 240 days; (**c**) age at 60 °C for 240 days; (**d**) age at 70 °C for 240 days.

The aging behavior of PBT binders and their elastomers is also one of the key directions in the polymer binder research community. In practical applications, PBT binders and their elastomers are subjected to prolonged exposure in complex environments for a long time, such as high temperature, high humidity, ultraviolet radiation and other factors, which will cause their performance to deteriorate to varying degrees. To investigate the aging behavior of PBT binders deeply, to clarify its aging mechanism and influencing factors, to improve the stability of PBT binders and its propellants, and to expand its application range has an indispensable significance. Chen et al. [43] systematically studied the microscopic mechanism of PBT prepolymer and its elastomer based on BAMO/THF (1:1) copolymerization under accelerated thermal aging at 70 °C. The prepolymers exhibited marked chromatic darkening (Figure 8), increased fluidity, and reduced relative viscosity after 14 days of aging, indicating improved molecular chain motility and impaired structural integrity. XPS analysis showed that no C1sC peak was detected in the elastomer at the early aging stage, which was consistent with the initial crosslinked network structure. The increase in the C1sA peak and the decrease in the C1sB peak after 240 days of aging revealed the accumulation of oxidation products and the recombination of chemical bonds (Figure 9). The analysis demonstrated that the thermal aging of the PBT system is dominated by the oxidation of hydroxyl termini, oxidative crosslinking between macromolecular chains and degradation of chain breaking. Then, the active oxide produced by BDNPA/F decomposition leads to interchain crosslinking. Finally, the main chain of polymerization was irreversibly degraded. It is worth noting that the addition of radical scavenger BHT can significantly inhibit the thermal oxygen aging reaction, which proves that the active free radical scavenging strategy exerts a pivotal influence on improving the stability of the material.

**Figure 10:**
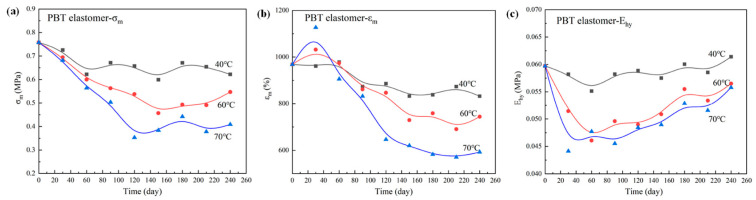
Change in mechanical properties vs. time: (**a**) *σ_m_*; (**b**) *ε_m_*; (**c**) *E_hy_* [44].

Meanwhile, Chen et al. [44] systematically investigated the thermal aging behavior of PBT elastomer at 40 °C, 60 °C and 70 °C, demonstrating a progressive decline in mechanical properties with extended aging time and elevated temperatures (Figure 10). The results show that the thermal oxidation process of PBT elastomers is characterized by multiple coupled mechanisms: the initial stage is dominated by hydrogen bond cleavage, then the degradation of polyurethane segments is intensified, and finally the oxidation crosslinking of polyether backbone becomes the main reaction path. Gas product analysis revealed that CO_2_ and N_2_O were derived from the main thermal aging gas products produced by polyurethane degradation and BDNPA/F decomposition, respectively, which confirmed the synergistic effect of chemical crosslinking network destruction and microphase separation reduction. Combined with the microstructure characterization, it is found that the hydrogen bond breakdown and polyurethane degradation lead to the increase in free volume and the decrease in crosslinking density, which are the key reasons for the decrease in macroscopic mechanical properties. This study provides theoretical support for the optimization of long-term storage stability of PBT-based binders in solid propellants, and it emphasizes the need to enhance the anti-aging properties by enhancing hydrogen bond strength and inhibiting oxidative crosslinking, which has significant guidance for the engineering application of high-energy, low-sensitivity propellants.

Researchers worldwide have extensively investigated the thermal properties, combustion properties, curing behavior, rheological properties, mechanical properties and aging properties of PBT binders. Through a series of experiments and theoretical studies, the regulatory mechanism of the multi-scale properties of these binders have been elucidated. However, the evolution rules of properties under complex environments (such as high pressure and multi-factor coupling) still need to be further explored. The synergistic effect and aging inhibition strategy of the composite system provide a new idea for the design of high-performance binders, which can be combined with molecular dynamics simulation and experimental verification in the future to further optimize its application performance in propellants and extreme environments.

## 3. Study on PBT-Based High-Energy Solid Propellants

PBT binder has been widely studied by scholars in the field because of its high energy, low glass transition temperature, adjustable mechanical properties at low temperature, controllable burning rate and low characteristic signal. The energy performance, technological performance, mechanical performance, combustion performance and safety performance of PBT propellants will be summarized.

### 3.1. Study on Energy Performance of PBT Propellants

Solid propellants are the power sources of solid rocket engines, and their energy characteristics determine the engine performance. The energy characteristics of propellant (such as specific impulse and characteristic speed) are one of the important factors in the selection and design of propellant formula. Based on the technical and mechanical properties of propellants, it is one of the goals of propellant researchers to continuously improve the energy level of propellants [45].

Significant advancements have been made in the research of energy enhancement of PBT high-energy solid propellants, but there are still challenges whereby the theoretical specific impulse is limited due to the low combustion temperature. Fan et al. [46] systematically evaluated the energy characteristics of PBT-based high-energy propellants with a plasticizer-to-binder ratio of 1.5 and 80% solid loading. Their results demonstrated that formulations containing 16 wt% Al and 39 wt% HMX achieved a theoretical *I_sp_* of 2724.2 N·s·kg^−1^, which was comparable to the energy level of GAP-based propellants. However, the combustion chamber temperature (*T_c_*) of the PBT system is significantly lower than that of the GAP-based propellants, resulting in a relatively limited theoretical specific impulse. This phenomenon is due to the high-density distribution of azide groups (-N_3_) in the PBT molecular chain, which gives them advantages of a high enthalpy of formation and low molecular weight gaseous products, but it reduces the exothermic efficiency of the combustion reaction. The study further revealed that the energy characteristics of PBT-based propellants can be improved by optimizing the synergistic use of oxidants and high-energy fillers, which provides a key theoretical basis for the design of solid propellants with both high energy and process stability. The results highlight the potential of PBT binders for high-energy propellant applications and emphasize the need to overcome their inherent thermodynamic limitations by regulating the combustion dynamic balance.

**Figure 11:**
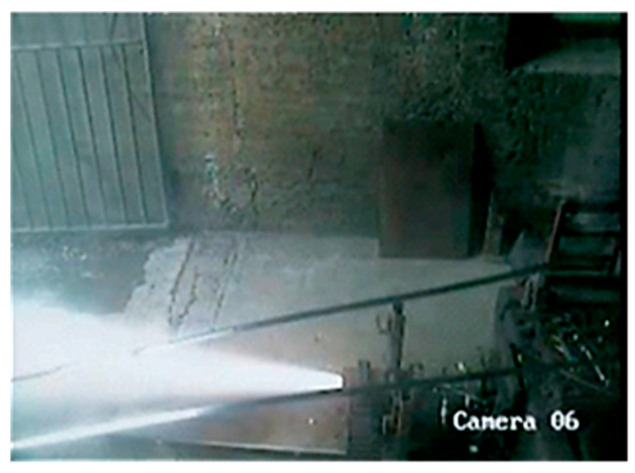
Standard engine test diagram for 5% Al and 73% AP [47].

To further improve the energy characteristics of PBT high-energy solid propellants, Wu et al. [47] successfully developed a new solid propellant with both low smoke and high energy by optimizing the formulation system of PBT-based three-component propellants (78% solid content, 5% aluminum powder). The results show that by using the synergic action of composite plasticizer (ATC) and combustion catalyst (RC), the measured specific impulse of the propellant is 246.4 s, the density is 1.764 g·cm^−3^, and the pressure index is significantly reduced to 0.36 (7–15 MPa range) under the working condition of Ø118 standard engine at 7 MPa (Figure 11). By adjusting the amount of bonding agent (0.15% mass fraction) and curing parameter (0.95), the formula achieves good mechanical properties over a wide temperature range (high-temperature tensile strength 0.709 MPa, low-temperature elongation 52.0%). In addition, the molar fraction of Al_2_O_3_, CO and H_2_ in combustion products decreased significantly (by 60%, 59% and 85%, respectively). The study confirmed that PBT binder can take into account the energy release efficiency and environmental friendliness through the high-density distribution of azide groups and the synergistic ratio of oxidant/catalyst in the system with low aluminum content, which provides an important technical path for the engineering application of low characteristic signal propellants.

**Figure 12:**
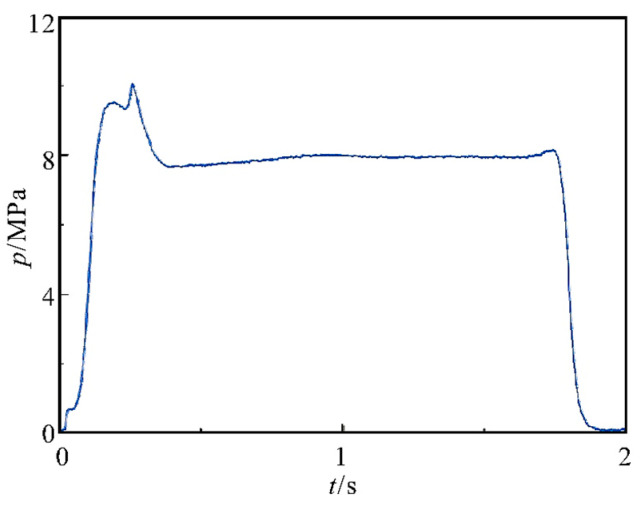
The p-t curve of PBT propellant ground test [48].

Zhang et al. [48] used PBT as binder and A_3_ as plasticizer, and they studied PBT-based low-smoke propellants by using HMX to partially replace oxidant AP and reducing the amount of propellant Al, finding that the PBT low-smoke propellant exhibited low slurry viscosity and good process performance. When the dosage of bonding agent is 0.3% and the curing parameter is 1.3, the high-temperature tensile strength of the propellant is 0.511 MPa, and the elongation at room temperature, high temperature and low temperature is greater than 40%. The BSFØ165 engine charging and ground test have been completed. The results show that the p-t curve is normal and measured at 6.86 MPa (Figure 12). Notably, the propellant met reduced-smoke criteria, with visible, infrared, and laser transmittance exceeding 70%, underscoring its potential for high-energy, low-signature applications in advanced solid rocket motors. This work provides critical insights into the synergistic design of binder systems and formula optimization for next-generation propulsion technologies.

To sum up, the current research on PBT propellants has achieved a balance between energy output and process performance through formulation optimization and process improvement, but it is necessary to further overcome the combustion temperature limit to improve the theoretical specific impulse of the system.

### 3.2. Study on Technological Performance of PBT Propellants

**Figure 13:**
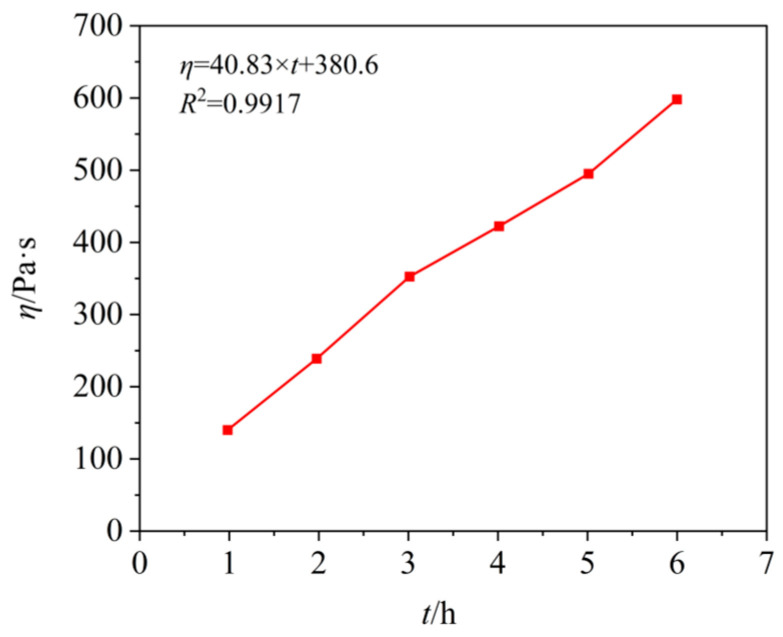
The relation between viscosity of PBT propellant slurry and time [48].

The excellent performance of solid propellants directly affects the quality of solid motor charge. Therefore, good fluidity, the leveling of the propellant slurry, and the long service life of the charge are the keys to judging the technical performance of propellants. Zhang et al. [48] have studied the viscosity of the PBT-based three-component propellant slurry using a falling ball viscometer (Figure 13). The test results show that the viscosity of the PBT propellant slurry is 416 Pa·s after 1 h of discharge, and 626 Pa·s after 6 h. The viscosity increases slowly with time, thus meeting the technological requirements that the viscosity of the PBT propellant slurry is less than 1500 Pa·s after being discharged for 6 h, which shows that it has excellent service life and controllable viscosity growth characteristics. This is because the solid content in PBT-based low-smoke propellants is low, the solid grading is reasonable, and the dual curing system (LM-100/IPDI) is used. By regulating the reactivity, the double-curing system significantly delayed the viscosity rise rate of the slurry, thus ensuring the fluidity and operability of the slurry.

It can be seen that the PBT-based propellant has successfully achieved the improvement of process performance through the collaborative optimization of the formulation and curing system, which provides technical support for high-reliability solid motor charging. Future studies can further explore the fine regulation of solidification kinetics and solid phase distribution to take into account the dual requirements of process performance and energy characteristics.

### 3.3. Study on Mechanical Properties of PBT Propellants

Good mechanical properties are the basis for the realization of other propellant properties. Improving the mechanical properties of propellants and ensuring the integrity of propellant grain structure under various loads is the premise to achieve the combustion performance and safety performance of propellants. Moreover, improving the low- and high-temperature mechanical properties of propellants is meaningful to enhance the environmental adaptability of solid propellants [49,50]. Improving the mechanical properties of PBT-based high-energy solid propellants mainly starts from the following two aspects: one way is to improve the mechanical properties of the binder matrix itself, and the other is to strengthen the interface bonding between the binder matrix and the solid filler.

In a systematic investigation to optimize the mechanical performance of BAMO-THF propellants, Xie et al. [51] evaluated the effects of various curing agents, including HDI, TDI, IPDI, and N-100, on tensile strength and elongation properties. Their findings revealed that the multifunctional curing agent N-100 significantly enhanced the tensile strength of the propellant (reaching 0.734 MPa at 25 °C and 0.656 MPa at 50 °C) due to its high crosslinking density. However, this increased rigidity concurrently led to a marked reduction in elongation (21.2–29.9% across temperatures). In contrast, the difunctional curing agent TDI achieved a balanced performance, improving strength while maintaining superior elongation (50.3–54.7%), which was attributed to its moderate crosslinking that preserved network flexibility. These results underscore the critical role of curing agent functionality in tailoring the mechanical trade-off between strength and ductility, highlighting TDI as a promising candidate for propellant formulations requiring the synergistic enhancement of both properties. This insight aligns with advancements in binder systems, emphasizing the strategic selection of curing agents to optimize polymer network architectures in high-performance propellants.

In a comprehensive study aimed at optimizing the mechanical and thermal properties of PBT-based propellants, Li et al. [52] systematically investigated the roles of chain extenders (BDO, PET, PEG), crosslinking agents (TN-J, TMP, PTT, T-PEG), and plasticization ratios. Their findings revealed that PEG, as a macromolecular chain extender, significantly enhanced the maximum elongation (up to 75% at −55 °C) while maintaining tensile strength stability (0.6–0.7 MPa at 70 °C), which was attributed to its ability to increase inter-crosslink molecular weight and reduce network defects. Among crosslinking agents, TN-J exhibited superior performance in improving tensile strength (1.01 MPa at 20 °C), outperforming TMP and PTT, due to its dual functionality as a crosslinker and bonding agent. The cyano groups in TN-J formed hydrogen bonds with ammonium ions in AP, enhancing interfacial adhesion and network integrity. At an optimized plasticization ratio of 2.0, synergistic adjustments of PEG (0.1–0.3%) and TN-J (0.2–0.6%) achieved a balanced performance profile: high-temperature strength (0.6–0.7 MPa), exceptional low-temperature elongation (45–75%), and a suppressed glass transition temperature (less than 65 °C) (Table 3). These results highlight the critical interplay between the molecular design of binder and formulation optimization in tailoring PBT-based propellants for wide-temperature applications.

**Figure 14:**
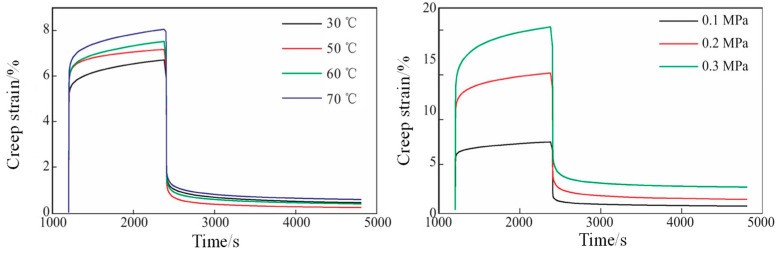
Creep mechanical properties of PBT propellants at different test temperatures (**left**) and under different loading stresses (**right**) [53].

To enhance the high-temperature mechanical properties of PBT-insensitive high-energy propellants, Hu et al. [53] investigated the mechanical properties of PBT propellants at high temperature through creep tests (Figure 14). The results showed that the high-temperature creep performance of PBT propellants is closely related to stress and temperature, and especially the load effect is remarkable. With the increase in curing parameter r, the matrix network structure can be improved and the mechanical properties of propellants can be improved as well; thus, the creep resistance of propellants can be improved. With the increase in plasticization ratio and the viscosity contribution of mechanical properties, and the creep degree and failure deformation increase, making the creep resistance of the samples decrease, while the samples with high plasticization show significant high-temperature softening phenomena.

Shen et al. [54] studied the high-temperature mechanical properties of the propellant by optimizing the amount of neutral bonding agent and the molecular weight of the curing network. The results showed that (1) the ethanolamine bonding agent lacks effective chemical bonding on the surface of HMX particles, and HMX can be partially dissolved in polar plasticizer A_3_, forming a soft interface layer, which made the surface of HMX easy to dehumidify and affected the high-temperature mechanical properties of propellants. (2) By adjusting the amount of crosslinking agent and curing parameters, the curing network can be effectively optimized, and the PBT-insensitive high-energy propellant formula with excellent mechanical properties at different temperature environments can be obtained by combining the composite bonding agent technology. (3) When the crosslinked molecular weight reached 8000–10,000 and the amount of neutral bonding agent NPBA was 0.08–0.10%, the high-temperature tensile strength of the propellant was more than 550 KPa, and the maximum elongation rate could reach more than 40%. Zhang et al. [55] studied the effects of curing parameters, crosslinking parameters, curing time and other parameters on the mechanical properties of PBT propellants at low temperatures. The results showed that when curing parameter was 1.02 and crosslinking parameter was 0.8, the comprehensive mechanical properties were better, and at the low temperature of −40 °C, the propellant *σ_m_* was positively correlated with curing parameters and crosslinking parameters, while the propellant *ε_m_* was negatively correlated with curing parameters and crosslinking parameters.

In addition, in the study of the interface mechanical properties between the PBT matrix and AP filler, domestic scholars [25] systematically revealed the synergistic mechanism of the PBT matrix crosslinking degree and AP surface defects on propellant interface adsorption behavior by combining molecular dynamics simulation methods. The results show that the fully crosslinked (90%) PBT matrix enhances the interface binding energy through the rigid network structure, while the incomplete crosslinked (80%) matrix weakens the interface adsorption strength due to the lack of functional groups. The 30 *Å* defect on the AP surface significantly improves the adsorption strength by enlarging the contact area, while the excessive defect (40 Å) leads to the deterioration of the interface mechanical properties due to stress concentration. This study elucidates the interfacial adsorption mechanism led by hydrogen bond and van der Waals force at the atomic scale, which provides a key theoretical support for the crosslinking network design and defect optimization of PBT azide propellants with high debonding resistance.

Simultaneously, researchers [56] have systematically elucidated the stress relaxation behavior and regulatory mechanisms of BAMO-THF-based azido propellants under elevated temperatures by integrating dynamic mechanical analysis (DMA) with microstructural characterization. Experimental results demonstrate that the four formulated propellants exhibit elastomeric composite behaviors, which are characterized by pronounced stress relaxation at the initial stage and significant thermal susceptibility. Increasing the plasticizer-to-polymer mass ratio (*p_l_*/*p_o_*) to 1.2 enhances the overall elongation of the propellant but compromises its tensile strength, which is attributed to the lubricating effect of plasticizers on polymer chains. Furthermore, the synergistic use of diamine (MOCA) and diol (BDO) chain extenders improves low-temperature mechanical performance, albeit inducing marked stress relaxation at high temperatures. This study proposes that optimizing the ordering degree of crosslinked networks or enhancing their structural integrity effectively suppresses high-temperature stress relaxation. These findings provide a strategic pathway for advancing the thermal resistance and mechanical stability of azido propellants in high-energy applications.

The mechanical properties of propellants are the core basis to ensure their combustion performance, safety performance and environmental adaptability. The optimization focuses on the two directions of improving the properties of the adhesive matrix and strengthening the filler–matrix interface bond, including the regulation of the curing system and the compounding of bonding agents, etc., and it establishes a more systematic regulatory framework for mechanical properties. However, how to achieve the dynamic balance of strength and elongation at extreme temperatures and restrain the interface deterioration caused by plasticizer migration still needs to be further explored. Future research can focus on the development of new bonding agents, multi-scale network structure design and dynamic mechanical response mechanism analysis so as to promote the engineering application of PBT propellants in complex service environments.

### 3.4. Study on Combustion Performance of PBT Propellants

To ensure that the solid propellant possess brilliant and stable combustion performance under working conditions, the combustion performance of solid propellants is adjusted and optimized from various aspects (formula composition, particle gradation and burning rate regulator) to effectively control the energy release rate of propellants and ensure the weapon gains stable interior ballistic performance. In general, the parameters that characterize the combustion characteristics of solid propellants mainly include burning rate, pressure exponent, burning rate temperature coefficient, and so on.

Yu et al. [57] reported a new PBT propellant with ammonium dinitramide (ADN) as a high-energy oxidizer. It was found that the burning rate of the propellant increased from 7.9 mm·s^−1^ to 117.4 mm·s^−1^ when the pressure increased from 0.1 MPa to 10 MPa, and it increased from 49.1 mm·s^−1^ to 74.2 mm·s^−1^ when the pressure increased from 12.0 MPa to 20.0 MPa. The combustion process of the ADN/PBT propellant changed from diffusion control reaction to kinetic control reaction under the oxidant load of 0.1–10 MPa and 60 wt%–70 wt%. Meanwhile, it was found that the particle size of ADN had no significant effect on the combustion performance of the propellants, which may be due to the heat dissipation effect of the solid–liquid mixed multi-phase layer and PBT binder on smaller ADN particles. Song et al. [58] explored the impact of Cr powder, TiH_2_, carbonate, ammonium salt (TA), linear ammonium nitrate (DNP), TATB, nitroguanidine, ammonium nitrate (AN) and other substances on the burning rate of PBT propellants. Through the test results of the Ø118 engine, the pressure index of PBT propellants containing TA and carbonate was 0.43 (*p* = 5.544). Wu et al. [47] designed a PBT-based three-component propellant formula containing 5% Al to obtain a composite solid propellant with less smoke and high energy. The experiment exhibited that the pressure index of the propellant decreased gradually with the increase in the content of plasticizer (ATC) and burning rate copper–chromium catalyst (RC), but the burning rate of the propellant increased with the increase in RC and ultrafine ammonium perchlorate AP. Finally, considering the energy level and pressure index performance of the propellant, when an ATC of 2.4% and RC of 0.2% are used, the best propellant formula with the pressure index of 0.36 is obtained. Wang et al. [59] carried out a systematic study on the formulation design of boron-containing BAMO/THF (1:1)-based solid propellants and the collaborative optimization of energy–mechanical properties. By adjusting the mass ratio of the plasticizer/binder, solid content and HMX/AP/boron composite filler system, the propellant with a low burning rate (4.79 mm·/s^−1^ at 7 MPa), low pressure strength index (*n* = 0.30) and wide range burning rate regulation was successfully prepared. The experimental results show that the system has a specific impulse efficiency of 84.45% in the test of a Ø65 mm engine, which verifies its energy compensation advantage in the environment related to the high expansion ratio of a monolithic engine. The mechanical properties test shows that the tensile strength at room temperature reaches 0.7 MPa, while the elongation at low temperature (−40 °C) is 19.61%, indicating that the interface compatibility between the BAMO/THF adhesive network and boron-containing filler is good. This study highlights the core role of adhesive system design and multi-phase component optimization in improving the overall performance of propellants.

The optimization of solid propellant combustion performance is the core problem to achieve the stable internal ballistic performance of weapon systems. The existing research shows that the energy release and combustion stability can be effectively balanced through the composition control, the optimization of a burning rate regulator and the cooperative design of a multi-phase system. These studies show that the functional design of components based on the combustion mechanism, interface engineering of a multi-phase system and gradient matching of a burning rate regulator are the key directions for the improvement of propellant performance in the future.

### 3.5. Study on the Safety Performance of PBT Propellants

With the use of modern high-performance weapon platforms, the requirements for the energy performance of propellants are increasing. The energy and safety of propellants are a pair of contradictory constraints: that is, the higher the energy, the higher the sensitivity, and the lower the safety [60]. Therefore, reducing the sensitivity of propellants and improve their safety performance is of great significance.

Aiming at the safety performance optimization of PBT-based propellants, Wu et al. [61] studied the influence mechanism of HMX (10–20%) and Bu-NENA (10–15%) contents on the danger level. It was found that the introduction of Bu-NENA significantly deactivated the mechanical sensitivity of the propellant, and the synergistic effect between the flexible butyl segment and nitramine/nitrate group in its molecular structure effectively reduced the impact sensitivity of the PBT adhesive system. The critical threshold experiments show that when the HMX content is less than 13% and the Bu-NENA content is less than 12%, the propellant risk level can be optimized to 1.3 (according to GJB 6195-2008 standard), while maintaining the high-energy characteristic of a theoretical specific impulse ≥ 267 s. The system showed excellent mechanical adaptability over a wide temperature range (−60–70 °C). The balance between the glass transition temperature (Tg = −65 °C) and the viscoelastic energy proved that the Bu-NENA/PBT binder network enhanced the interfacial compatibility at extreme temperatures. The study further reveals the key value of the molecular design of an energetic plasticizer and PBT binder for the multi-objective cooperative optimization of propellant “high energy, passivity, wide temperature adaptation”, which provides theoretical support and a technical path for the safety and reliability improvement of tactical weapon propulsion systems.

Wang et al. [62] investigated the effect of PSAN on the low vulnerability of PBT-based solid propellants by replacing AP with insensitive oxidant PSAN based on ensuring that the properties of the sample formula did not change obviously. The results show that when PSAN completely replaces AP, the reaction levels of the samples in a slow-burning test, sympathetic explosion test, and jet impact test are explosion, detonation and combustion, respectively. When PSAN was used to replace part of AP, the reaction temperature of the sample decreased from 163.2 °C to 128.6 °C in a slow baking test, and the reaction level changed from explosion to combustion, lower than explosion and combustion in the jet impact test and sympathetic explosion test, respectively, indicating that the addition of PSAN can effectively improve the low vulnerability of PBT-based solid propellants.

**Figure 15:**
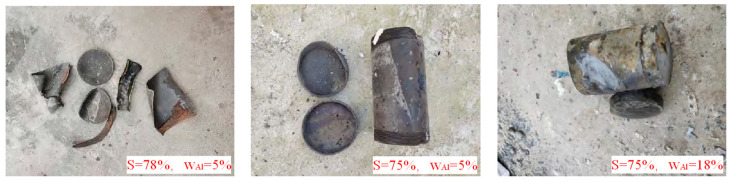
The effect of solid content and Al on response to slow roasting composition changes on the response of slow cook-off [63].

In a complementary study, Li et al. [63] investigated the influence of different solid content and Al content on the slow-burning response of PBT propellants (Figure 15), and they found that the burning response temperature was 158.6 °C when the solid content was 78% and Al content was 5% and the burning response was deflagration. Notably, reducing solid content to 75% while maintaining Al loading (5% or 18%) eliminated explosive responses, exhibiting only partial combustion chamber rupture. These findings demonstrate that reducing the solid content is beneficial to the propellant, reducing the slow-baking response.

Based on the current research progress of the safety performance optimization of PBT-based propellants, different component control strategies show significant synergistic effects on reducing risk grade and improving low vulnerability. In the future, the safety performance of PBT propellants should be improved through multi-dimensional strategies such as the molecular design of energetic components, passivity substitution of oxidants, and cooperative optimization of multi-phase fillers, to provide a theoretical basis and engineering practice path for the development of PBT propellants with excellent comprehensive performance.

## 4. Conclusions

PBT is a promising energetic binder for solid propellants due to its high-energy output, insensitivity, low characteristic signal, and environmentally friendly characteristics. In this paper, the synthesis of a PBT binder is briefly introduced, and the PBT binder research related to its thermal performance, combustion performance, curing, rheology, mechanical performance and aging performance is summarized and analyzed. Meanwhile, the energy performance, mechanical performance, combustibility and safety performance of PBT propellants are summarized emphatically. However, it has been found that there is little research on the rheological properties and aging properties of PBT propellants. While PBT propellants exhibit excellent low-temperature mechanical properties, their energy density requires further optimization. Therefore, we can conduct in-depth research from the above two aspects in the future. Moreover, considering the mechanical and technological properties of the propellant, the energy performance level of PBT propellants can be further improved by introducing functional groups into BAMO, changing the structural form of BAMO-based polymers, and introducing high energy density materials into the system.

## Figures and Tables

**Table 1:** Physicochemical properties of typical PBT [30].

Parameters	Values
Mean molecular weight (g·mol^−1^)	2240
Density (g·cm^−3^)	1.27
Melting point (°C)	−27
Glass transition temperature (°C)	−61
Enthalpy of formation (kJ·kg^−1^)	1185
Adiabatic combustion temperature (°C)	851

**Table 2:** Mechanics parameters of PBT/N-100 films [42].

*R* Value	*σ_m_* (MPa)	*ε_m_* (%)
0.8	0.902	220
0.9	0.963	143
1.0	1.119	121
1.1	1.329	125
1.2	1.296	118
1.3	1.345	116
1.4	1.362	115
1.5	1.365	120
1.6	1.355	117
1.7	1.354	110

**Table 3:** Effect of chain extender and crosslinker content on mechanical properties of PBT propellants [52].

PEG (%)	TN-J (%)	20 °C		70 °C		−55 °C		*T_g_* (°C)
		*σ_m_* (MPa)	*ε_m_* (%)	*σ_m_* (MPa)	*ε_m_* (%)	*σ_m_* (MPa)	*ε_m_* (%)	
0.1	0.2	1.01	57.2	0.60	42.7	4.62	70.0	−68.94
0.1	0.3	1.12	44.3	0.73	37.5	3.90	45.0	−68.30
0.2	0.4	1.30	41.6	0.68	32.2	4.36	61.8	−65.59
0.3	0.2	0.90	67.5	0.61	46.2	3.67	73.9	−70.10

## Data Availability

No new data were created or analyzed in this study.
